# I rhyme / To see myself, to set the darkness echoing^1^

**DOI:** 10.3201/eid1806.AC1806

**Published:** 2012-06

**Authors:** Polyxeni Potter

**Affiliations:** Centers for Disease Control and Prevention, Atlanta, Georgia, USA

**Keywords:** art science connection, emerging infectious diseases, art and medicine, Gerard van Kuijl, Narcissus, I rhyme to see myself, To set the darkness echoing, iatrogenic Creutzfeldt-Jakob disease, about the cover

**Figure:**
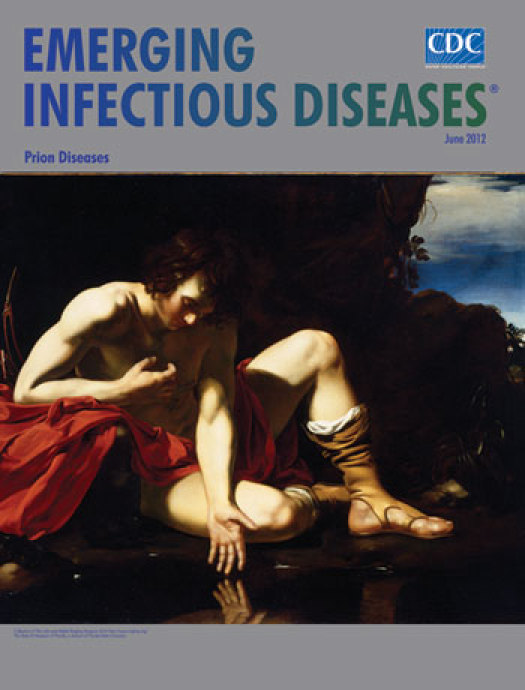
**Gerard van Kuijl, Dutch painter active in Rome (1604–1673) *Narcissus* (c. 1645) Oil on canvas (114 cm × 143 cm)** Collection of The John and Mable Ringling Museum of Art, http://www.ringling.org/ The State Art Museum of Florida, a division of Florida State University

“As a child, they could not keep me from wells,” wrote Seamus Heaney in his version of the Narcissus myth. “I loved the dark drop, the trapped sky, the smells / of waterweed, fungus and dank moss.” In countless versions, the ancient myth strikes a universal chord: person sees self, meets death. Ovid told of handsome Narcissus and Echo, the nymph who fell in love when she saw him “chasing frightened deer into his nets.” Rejected, she wasted away, until nothing was left but her voice, “heard by all.” He, “Tired from both his enthusiasm for hunting and from the heat,” rested a spell, caught glimpse of his reflection in a pool of water, and fell in love with “all the things for which he himself is admired.” Unable to tear himself from the fateful reflection, he too wasted away. At this spot later sprouted narcissus, the flower.

Favored by poet and artist alike, the story intrigues anyone who searches for or reconstructs the self. In art, variations abound, among them one by the great Michelangelo Merisi da Caravaggio (1571–1610). Contrary to the conventions of his age, he painted directly from posed models, a practice that cultivated a new relationship between painting and viewer by promoting art not as fiction but as an extension of everyday experience, the physical content enriched with psychological tension. Gerard van Kuijl, Dutch painter and follower of Caravaggio, might have seen the master’s *Narcissus* (1597) when he lived in Rome from 1629 to 1631.

In the 17th century, many artists from the northern Low Countries worked abroad or were influenced by others who had traveled and returned, bringing new styles to the local market. Their work strayed from the polders, woods, and dunes of the Dutch Golden Age to the biblical and secular, with human figures dominating the canvas. These artists exerted a lasting influence by introducing one of the main currents of baroque art into the Netherlands. The Caravaggists were mainly artists from Utrecht, who visited Italy and worked in the style of Caravaggio, characterized by realist drama and strong interplay of light and dark. This style also prevailed outside Utrecht, affecting Rembrandt and his followers.

In van Kuijl’s *Narcissus*, expert shading betrays Caravaggist influence as do the baroque shapes and style. In both the Caravaggio and van Kuijl renditions, the figure is wrapped in a mystical, isolating, introspective dark. But, despite the striking similarities, van Kuijl’s approach is individualized. While Caravaggio moved the figure into his own times showing no traces of classical attire, van Kuijl maintained topical decorum and a trace of the pastoral. Overall, it seems as if the two images represented a sequence. In the earlier painting the figure is actively engaged with his reflection, almost interactive, agile, embracing. In van Kuijl’s work, having given into the overwhelming attraction, the figure is entranced, dreamy, stochastic.

The theme of Narcissus is not new to science, having been exhaustively addressed in psychoanalysis and come down to us as narcissism and the narcissistic personality. In one iteration, the theme overlaps with the ever-popular myth of Pygmalion, the sculptor in antiquity, who fell in love not with his image but with his work―a female statue he created, one so perfect that it was, in his estimation, more beautiful than any woman could ever be.

Breathing life in or animating a work of art was not the domain alone of Pygmalion. Lyric poet Pindar in his seventh Olympic Ode wrote, “The animated figures stand / Adorning every public street / And seem to breathe in stone, or move their marble / feet.” Daedalus used to install voice in his statues, and Hephaestus created automata for his workshop. But these mechanical attempts with lifeless objects pale before more recent achievements, no less in modern medicine, which breathe life into dying human bodies with grafts, transfusions, and transplants, extending their tenure and the resilience of the species.

“Man,” wrote Johann Wolfgang von Goethe in his novel Elective Affinities, “is a true Narcissus. He makes the whole world his mirror.” The philosopher’s interest was literary, an opportunity to unravel personal and social processes and interpret the meaning of human actions and events. He thought that, like the young Seamus Heaney, man could not pass up an opportunity “To stare, big-eyed Narcissus, into some spring.” Nevertheless, as an adult, the poet himself found his early fascination with the well undignified. “I rhyme,” he professed, “To see myself, to set the darkness echoing.”

Goethe had no way of knowing that the time would come when humans would literally be able to recreate that which so fascinated them as myth and metaphor—not only recreate their image in a poem or, like Pygmalion, in art but also in the flesh. For loving one’s creation is certainly easy to fall into, even in science. A more resilient human with healthier and longer lasting parts is the reflection we look for in the well, a reflection too of improved medical knowledge, expertise, and technology: properly used antimicrobial drugs; preventive screening; and growth hormone and dura mater grafts from cadavers, the bold modern equivalent of magic and automata in statues. Yet the echo we expect to hear from the darkness is often interrupted by emerging pathogens and often, unawares, by ourselves.

The history of medicine is filled with examples of unintended consequences. A concern since the time of Hippocrates, “to do no harm” is a continuing chapter with an abundance of contemporary examples. In this issue of the journal, reliance on a single class of antimicrobial drugs for treatment of some infections heightens our vulnerability to emergence of resistance, requiring more treatment options. Preoperatively acquired emerging pathogens complicate liver transplantation, a problem threatening to increase, despite adequate infection control measures. On the other hand, comprehensive current tallies of global incidence of iatrogenic Creutzfeldt-Jakob disease identified no new sources of disease, indicating that current practices should continue to minimize the risk until blood screening is validated for human use and suggesting that, despite setbacks that make that glimpse of perfect self fatal, science diligently applied can still set the darkness echoing.
